# Closed-Loop Control of Electro-Ribbon Actuators

**DOI:** 10.3389/frobt.2020.557624

**Published:** 2020-11-16

**Authors:** Richard Suphapol Diteesawat, Aaron Fishman, Tim Helps, Majid Taghavi, Jonathan Rossiter

**Affiliations:** ^1^Department of Engineering Mathematics, University of Bristol, Bristol, United Kingdom; ^2^Bristol Robotics Laboratory, Bristol, United Kingdom

**Keywords:** control, soft robotics, actuator, electrostatic, zipping, pull-in instability, electro-ribbon, dielectrophoretic liquid zipping

## Abstract

Electro-ribbon actuators are lightweight, flexible, high-performance actuators for next generation soft robotics. When electrically charged, electrostatic forces cause the electrode ribbons to progressively zip together through a process called dielectrophoretic liquid zipping (DLZ), delivering contractions of more than 99% of their length. Electro-ribbon actuators exhibit pull-in instability, and this phenomenon makes them challenging to control: below the pull-in voltage threshold, actuator contraction is small, while above this threshold, increasing electrostatic forces cause the actuator to completely contract, providing a narrow contraction range for feedforward control. We show that application of a time-varying voltage profile that starts above pull-in threshold, but subsequently reduces, allows access to intermediate steady-states not accessible using traditional feed-forward control. A modified proportional-integral closed-loop controller is proposed (Boost-PI), which incorporates a variable boost voltage to temporarily elevate actuation close to, but not exceeding, the pull-in voltage threshold. This primes the actuator for zipping and drastically reduces rise time compared with a traditional PI controller. A multi-objective parameter-space approach was implemented to choose appropriate controller gains by assessing the metrics of rise time, overshoot, steady-state error, and settle time. This proposed control method addresses a key limitation of the electro-ribbon actuators, allowing the actuator to perform staircase and oscillatory control tasks. This significantly increases the range of applications which can exploit this new DLZ actuation technology.

## Introduction

Soft robotics has the potential to enhance dexterous robotics tasks, such as gripping and locomotion, using structures that conform to variable and sensitive environments (Schmitt et al., 2018). While soft robotic actuators offer the high stroke and force-to-weight ratio required for such tasks (Bar-Cohen, 2000; Carpi et al., 2011), the position control of rigid actuators is generally more predictable, and better suited for complex tasks such as path planning and position sensing in sufficiently structured environments (Trivedi et al., 2008). Thus, a significant challenge for soft robotics is the development of control strategies that permit fast and complex motion within changing or unpredictable environments.

**Figure 1:**
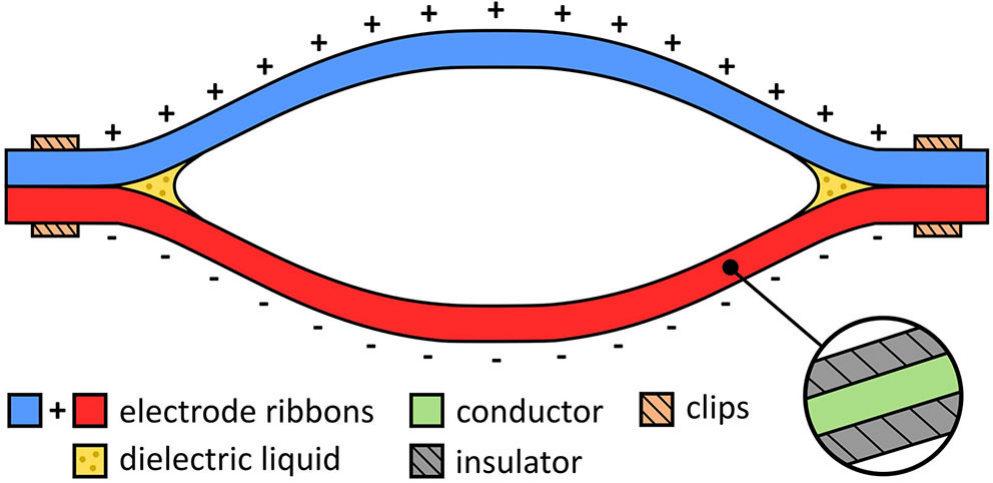
Components of an electro-ribbon actuator.

Dielectrophoretic Liquid Zipping (DLZ) is a novel actuation concept whereby amplified electrostatic attraction results in high-force electrostatic zipping (Taghavi et al., 2018). In DLZ, a pair of electrodes, electrically insulated from one another, are mechanically arranged in a zipping configuration (Figure 1). If the electrodes are oppositely charged, a strong electric field is developed between them, inducing a strong electrostatic attractive force that causes them to progressively zip together. Although electrostatic zipping has been demonstrated previously in zipping devices (Maffli et al., 2013; Chen et al., 2014; Li et al., 2018), DLZ features a tiny droplet of liquid dielectric (e.g., silicone oil) at the zipping point(s), which considerably amplifies electrostatic force. The electrostatic force between the two electrodes is related to Maxwell pressure, *P* = ε*E*^2^, where ε is the permittivity of the liquid dielectric and *E* is the electric field (Suo, 2010). The added liquid dielectric not only has a higher permittivity but also a considerably higher breakdown strength compared with air, allowing stronger fields to be sustained with associated stronger electrostatic zipping forces. Silicone oil, for example, whose respective permittivity and breakdown strength are around 2.7 and 6.7 times greater than air, theoretically implies up to 120-fold amplification of electrostatic force (Taghavi et al., 2018). While this amplification could be achieved by submerging the entire actuator in liquid dielectric, practically only a tiny droplet at each zipping point is required, since coincidentally occurring dielectrophoretic forces (which have the effect of drawing high-permittivity materials toward strong electric fields) help to retain the liquid dielectric at the zipping point (Taghavi et al., 2018).

The simplest embodiment of DLZ is the electro-ribbon actuator (Figure 1). Electro-ribbon actuators are compliant artificial muscles that can be made from any combination of conducting and insulating materials. Various embodiments of this type of actuator can exhibit high tension, high contraction (>99%), or high specific power equivalent to human muscle. These performance metrics make them a promising technology for Soft Robotics, where flexible, low-mass, high-performance actuators are required to deliver useful functions.

Control of soft robots is typically a challenging task due to their continuum structure and inherent compliance when interacting with the environment (Trivedi et al., 2008). Conventional control strategies that assume rigid joints tend to be ineffective at controlling soft robots (Rus and Tolley, 2015). Compared with traditional hard actuators, such as motors, control of soft actuators is often challenging due to their inherent compliance. Closed-loop control of dielectric elastomer actuators has been demonstrated using capacitive self-sensing (Rosset et al., 2013). Self-sensing has also been demonstrated in liquid-filled flexible fluidic actuators (Helps and Rossiter, 2018) and electrically driven HASEL (Acome et al., 2018) and Peano-HASEL (Kellaris et al., 2018) actuators, although full closed-loop control was not demonstrated. Closed-loop control of several parallel-plate electrostatic actuators, such as microelectromechanical systems (MEMS) devices, have been studied in Chu and Pister (1994), Seeger and Boser (1999), and Dong and Edwards (2010).

In this article, we investigate closed-loop control for electro-ribbon actuators. Open-loop control is limited with these actuators due to pull-in instability, resulting in an extremely small range of travel where position may be reliably controlled. However, by modulating the input voltage, a much larger region of stable positions can be achieved. We introduce a closed-loop (Boost-PI) controller, explore the effect of system gains upon output parameters, and configure gains based on a multi-objective parameter-space approach. Finally, we demonstrate system performance, including set point tracking of predetermined trajectories and sinusoidal signals, typical behaviors needed for the actuation and control of soft robots.

## Materials and Methods

**Figure 2:**
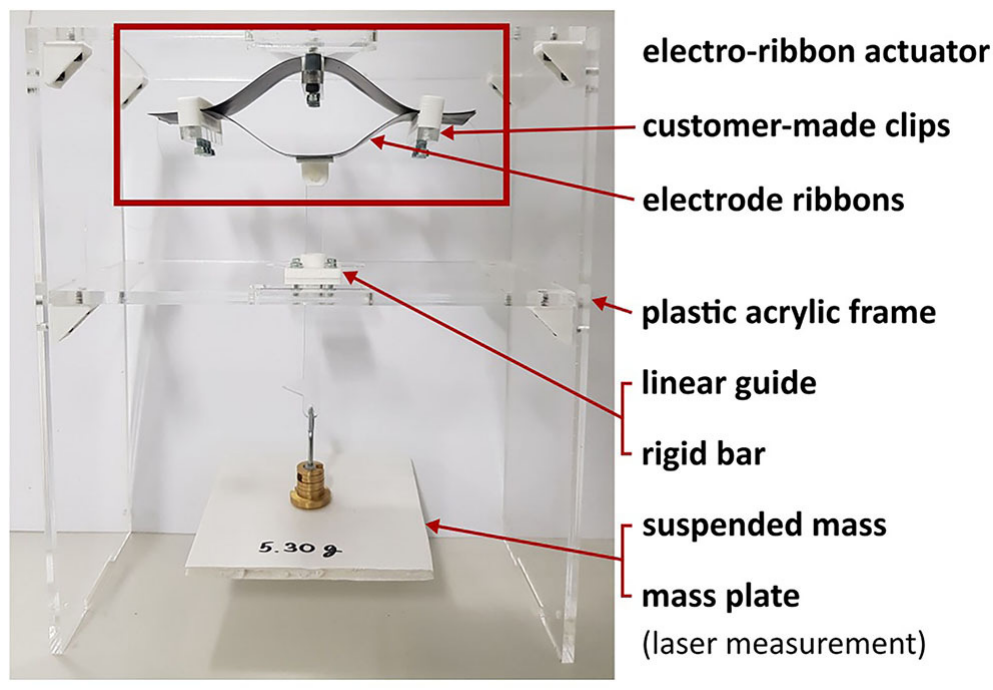
Experimental setup of the electro-ribbon actuator for both open-loop and closed-loop controls.

The electro-ribbon actuators were made from two electrode ribbons, each of which was comprised of a 50-μm-thick, 10-cm-long, 2.5-cm-wide steel strips (1.1274 carbon steel, h+s präzisionsfolien GmbH, Germany). Each electrode ribbon was insulated using PVC tape (AT7 PVC Electrical Insulation Tape, Advance Tapes, UK). The ends of the electrode ribbons were attached to one another using custom-made plastic clips to ensure a tight zipping point (Figure 2). A drop of silicone oil with viscosity of 50 cSt (# 378356, Sigma-Aldrich, USA) was added to each zipping point prior to each experiment to ensure consistency. High voltage was applied to the electro-ribbon actuators using a high voltage amplifier (5HVA24-BP1, UltraVolt, USA). Inputs were controlled and data was recorded using a National Instrument device (NI USB-6343, National Instruments, USA). A laser displacement sensor (LK-G402, Keyence, Japan) was used to measure actuator displacement, by measuring the height of the suspended mass in the vertical direction at a frequency of 1,000 Hz. For closed-loop control, measured height was used as the feedback variable, with a control loop sample frequency of 32 Hz. The open-loop bandwidth of the actuator has been observed as 10 Hz (Taghavi et al., 2018), thus 32 Hz was considered sufficient.

Isotonic testing was used to investigate the controllability of an electro-ribbon actuator. A rigid acrylic frame was built for the experiments. The center of the upper ribbon of the electro-ribbon actuator was clamped to the top of the rigid frame (Figure 2). This clamp had the advantage of preventing full zipping, which can introduce temporal hysteresis due to adhesive and cohesive forces associated with the liquid dielectric (Taghavi et al., 2018). The bottom ribbon was connected to a rigid bar, prescribed to move vertically by a linear guide, ensuring symmetrical zipping of the electro-ribbon actuator. To apply load to the electro-ribbon actuator, an external mass was hung at the bottom of the rigid bar. In the following experimental results, zero height was set as the position where the two electrodes zipped such that the bottom ribbon touched the clamp, with a 5 mm gap remaining between two electrodes at the center. The initial negative height was set as the resting position of the actuator with a suspended load and without an electrostatic force.

## Results

### Step Response

**Figure 3:**
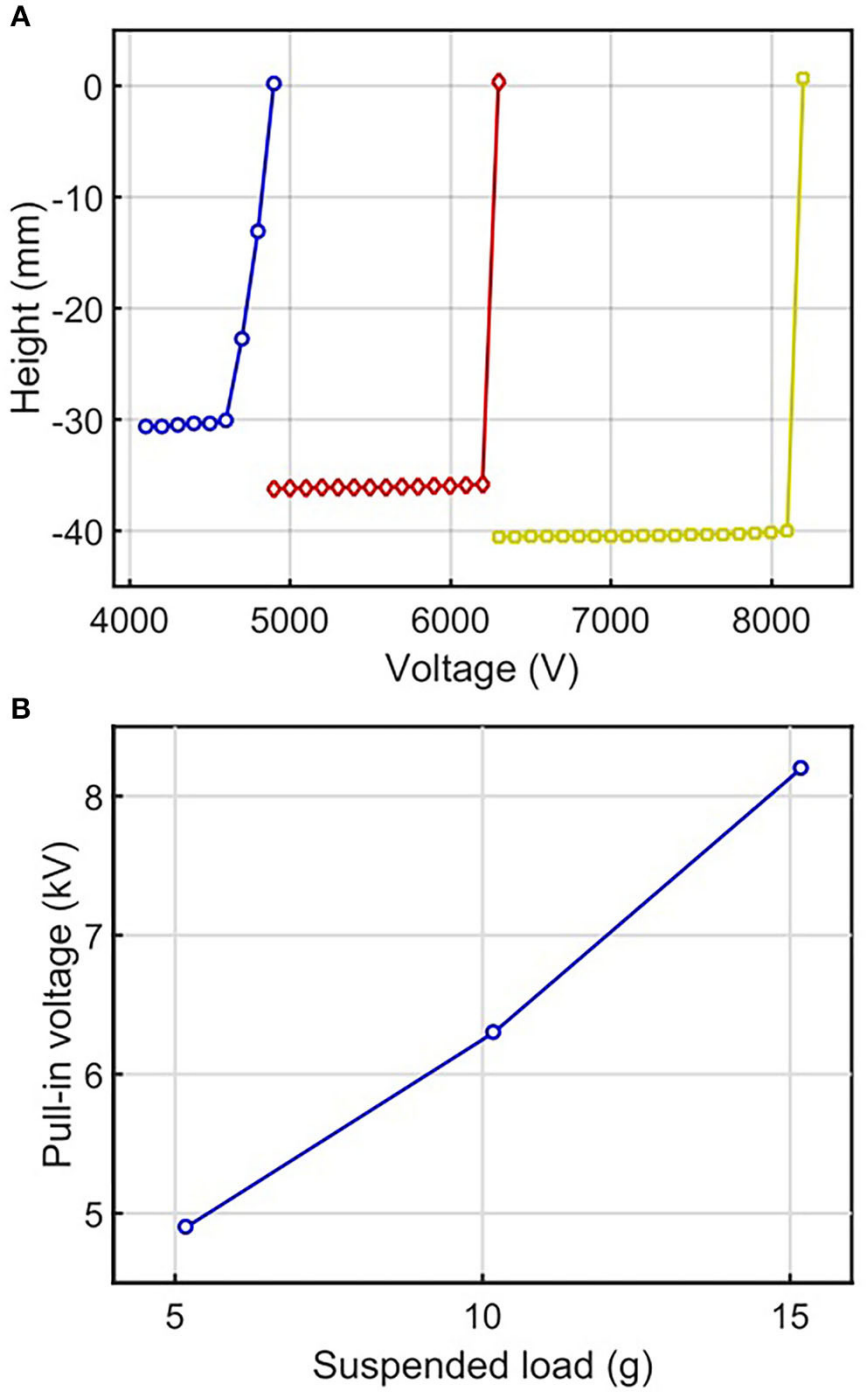
**(A)** Height variation with voltage for electro-ribbon actuators with masses of 5.18 g (blue line, circular markers), 10.18 g (red line, diamond markers), and 15.18 g (yellow line, square markers). Full zipping position occurs at zero height. **(B)** The relationship between pull-in voltage and suspended load.

To investigate the electrical charging effects, we performed a step-response test by applying a constant voltage, *V*_*constant*_, across the electrodes of the electro-ribbon actuator. In each test a constant mass was suspended from the actuator, which set its resting height. A constant voltage was applied for actuation. The vertical motion of the mass was recorded. After 10 s, the voltage was reduced to zero, and the actuator extended due to gravity, returning to its initial resting height for the next test at a higher voltage. The applied voltage was increased in 100 V increments for each test until pull-in voltage was reached. The maximum vertical displacement of the actuator achieved during 10 s of actuation at each voltage, was recorded and presented in Figure 3A. For example, for the actuator loaded with a constant mass of 10.18 g, when the *V*_*constant*_ reached 6,300 V, pull-in instability resulted in the actuator undergoing full zipping (Figure 3A). In this case, pull-in voltage *V*_*pull*−*in*_ was 6,300 V. This pull-in instability causes rapid full zipping because the generated constant electrostatic force at active moving zipping points consistently and increasingly overcomes the gravity force transferred from an external load, which is highest at zipping corners and decreases along the actuator to the center. As a result, when the electrostatic force at the zipping corners exceeds the gravitational load, the actuator will always fully zip. Figure 3A demonstrates how traditional open-loop control strategies for electro-ribbon actuators provide a very small controllable range. Since greater loads applied to the actuator require greater electrostatic force to initiate full zipping, *V*_*pull*−*in*_ increases with an external load. The relationship between pull-in voltage and load can be shown in Figure 3B.

### Time-Varying Voltage Profiles

**Figure 4:**
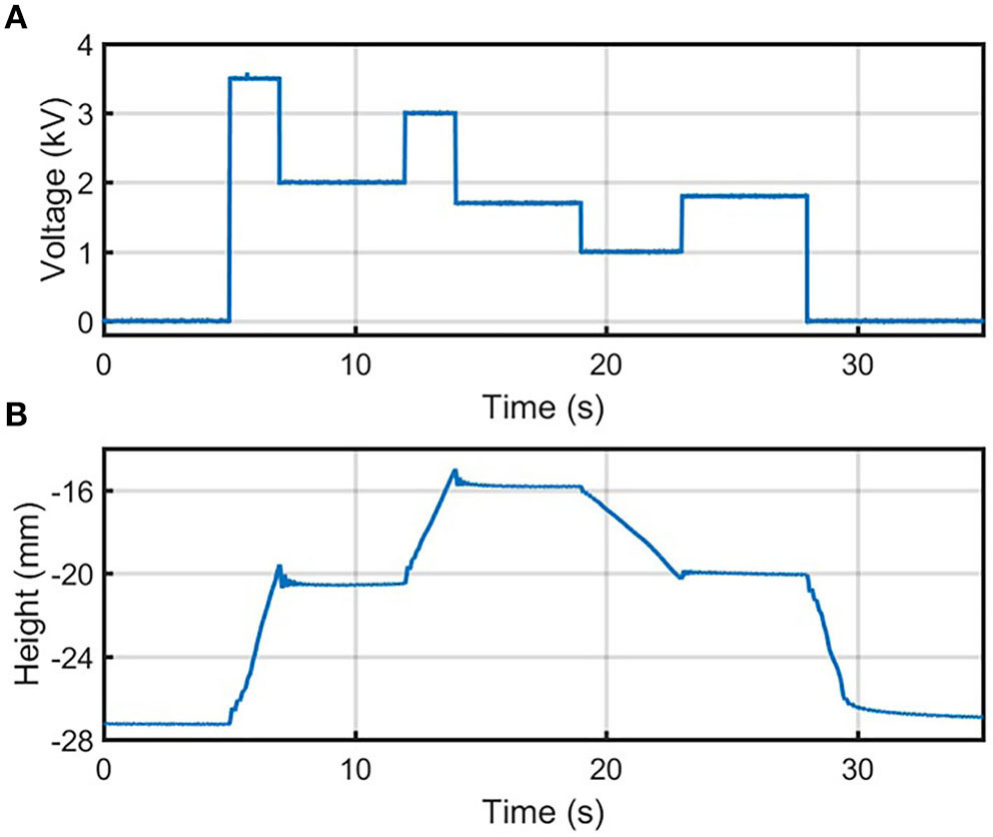
**(A)** The voltage and **(B)** height output of the time-varying voltage profile under a load of 7.05 g. Full zipping position occurs at zero height.

A more complex approach was explored by applying a time-varying voltage profile to the actuator (Figure 4A). In this experiment, the actuator began to zip when applying *V* ≥ *V*_*pull*−*in*_. After some time, *V* was instantaneously reduced to a constant value below *V*_*pull*−*in*_, which allowed the actuator to be held at a steady state height greater than the pull-in height in the previous step-response experiment. By switching *V* to a higher or lower value for a short time, and then setting a new constant voltage below *V*_*pull*−*in*_, we were able to move the actuator's position to multiple heights not accessible in the step-response experiment (Figure 4B).

For example, as shown in Figure 4B, the voltage is increased from 2 to 3 kV and thus contracts the actuator by roughly 5 mm. After decreasing the voltage to 1.7 kV, this steady-state position is maintained. The existence of additional stable heights within the range at which pull-in voltage occurs is attributed to various effects not included in standard pull-in instability models. These effects include stiction forces, and fluidic forces such as surface tension. These effects can be exploited to extend the contraction range of open-loop controllers of the electro-ribbon actuators. In addition, electro-ribbon actuators exhibit voltage-displacement hysteresis due to the inverse square relationship between actuation force and displacement at a given voltage (Taghavi et al., 2018). This hysteresis has been studied in detail in Taghavi et al. (2020).

### Closed-Loop Control

**Figure 5:**
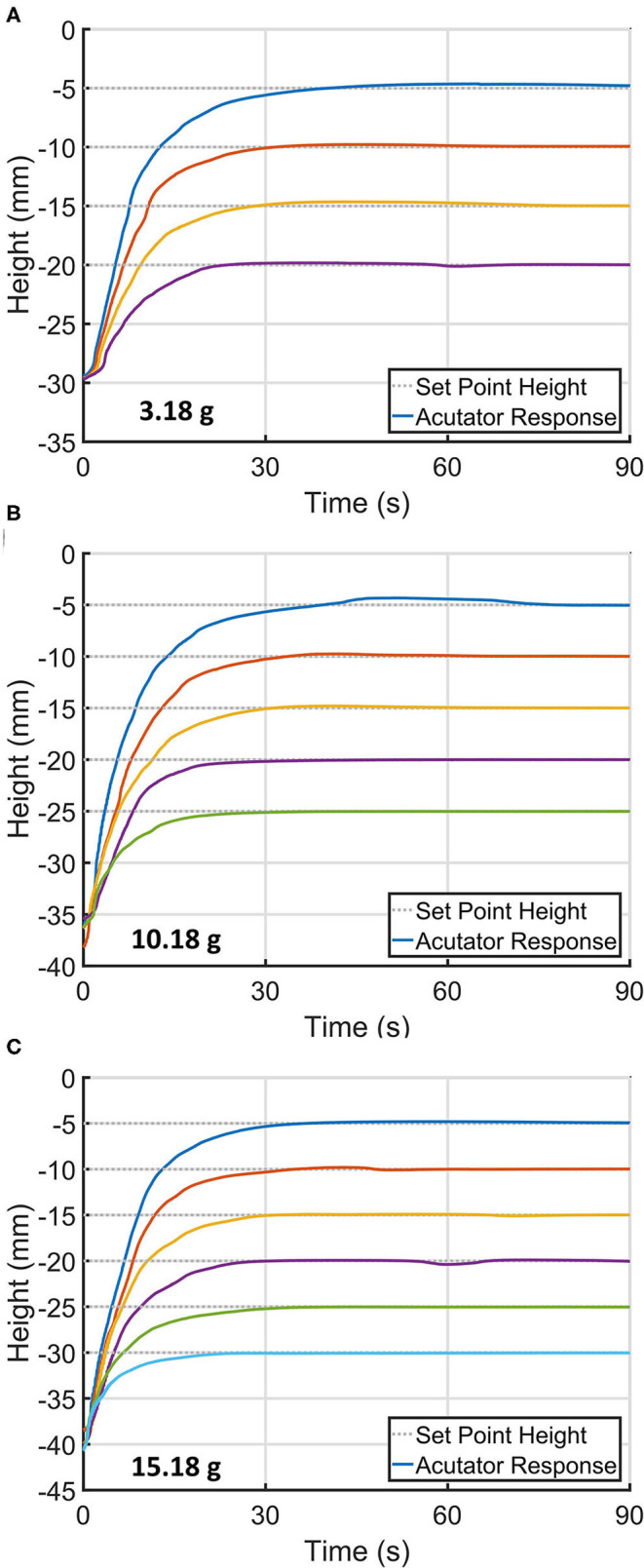
Time-series of the electro-ribbon actuator approaching different set point heights when using a simple controller with fixed proportional and integral terms of 600 and 60, respectively, while being loaded with different masses of **(A)** 3.18 g, **(B)** 10.18 g, and **(C)** 15.18 g.

Having found evidence of complex non-linearities affecting actuator stability within the system, we investigated a closed-loop controlled actuation by introducing a simple proportional-integral control. When using fixed proportional and integral terms of 600 and 60 respectively and setting the voltage to initialize at *V*_*pull*−*in*_, the actuator controllably approached to different set points while loaded different masses of 3.18, 10.18, and 15.18 g (Figure 5). This method allowed the electro-ribbon actuator to access to different intermediate heights between resting and full zipping points. However, an actuation speed decreased when set point height was set further, and the actuator suffered from long rise time to reach the desired height. Based upon these findings, we developed a modified closed-loop proportional-integrator (PI) controller, termed the Boost-PI controller. The control law took the form of the following equations:

(1) $$\begin{array}{r} E\left(t\right)\;=\;h\left(t\right)-h_{s}, \end{array}$$

where *E* is the input error, which is the difference between the measured height *h* and the setpoint height *h*_*s*_ of the electro-ribbon actuator. The input voltage *V* can be derived as follows when *t* is time:

(2) $$\begin{array}{r} V\left(t\right)\;=\;K_{p}E\left(t\right)\;+\;\int K_{i}E\left(t\right)dt\;+\;V_{c}. \end{array}$$

The Boost-PI controller was used to calculate the input voltage *V*, consisting of three parts as follows:

- *K*_*p*_: a proportional term, providing a large initial voltage proportional to the error. This term acts to rapidly initiate zipping.
- *K*_*i*_: an integral term, which acts to minimize steady-state error and to compensate for variable external loads.
- *V*_*c*_: a constant voltage equal to 90% of *V*_*pull*−*in*_, the voltage at which the actuator overcomes the load and begins to contract. This term acts to prime, or boost, the actuator for zipping, reducing rise time.

The voltage applied to the electro-ribbon actuator needs to exceed *V*_*pull*−*in*_ in order to initiate zipping. If only proportional and integral terms are used (standard PI controller), the actuator experiences large integration timescales, causing a long delay to reach this critical voltage. For example, if the proportional term is much lower than *V*_*pull*−*in*_, the integral term will take long time to accumulate until the total input voltage *V* reaches *V*_*pull*−*in*_. In this regard, *V*_*c*_ is set as 90% of *V*_*pull*−*in*_, ensuring the actuator is immediately almost at the point at of zipping. We also limit the maximum voltage applied to the actuator to 9,000 V, to prevent damage to the actuator or electric breakdown of the nearby air.

**Figure 6:**
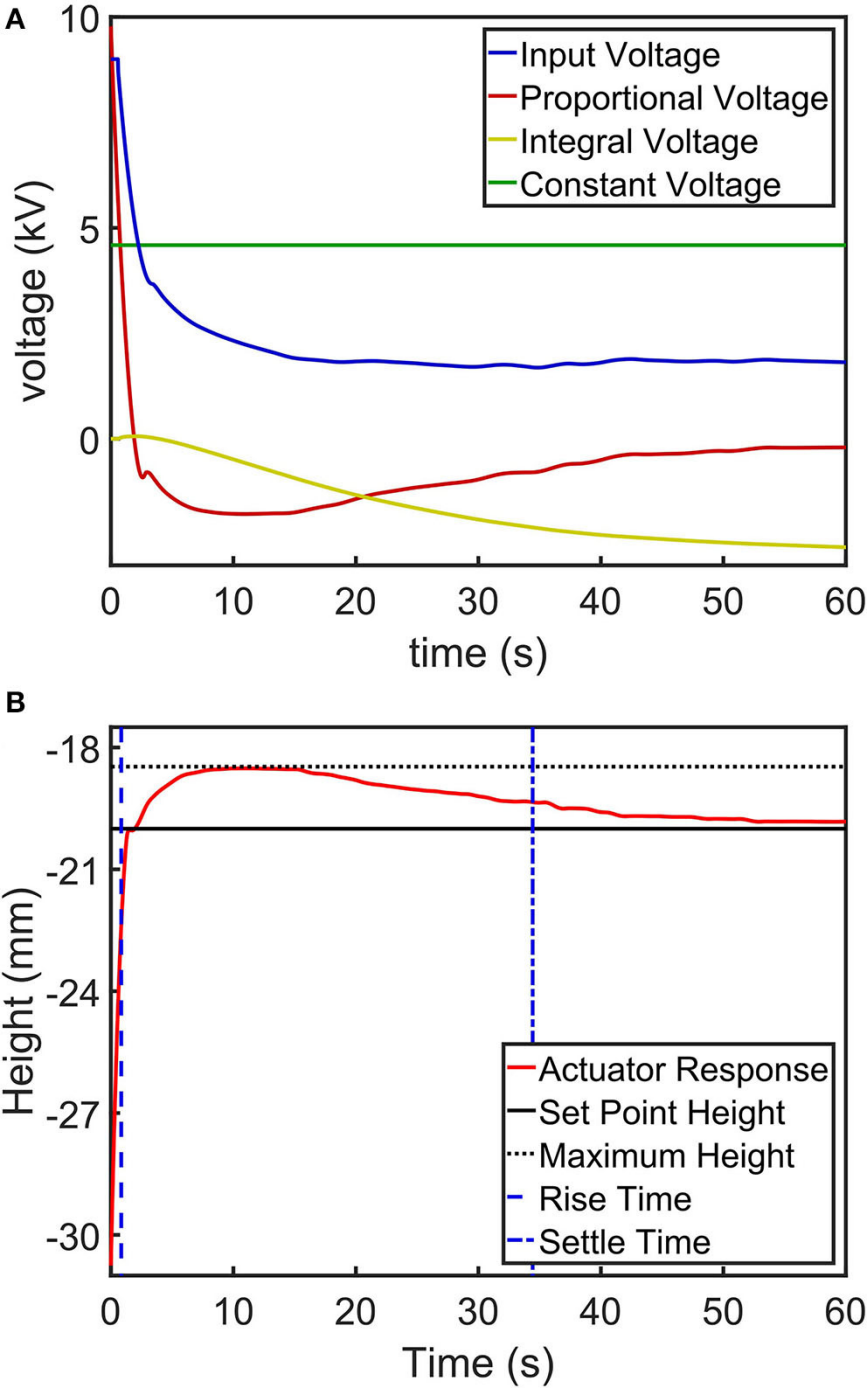
An example step-response control task of an electro-ribbon actuator actuated using a closed-loop Boost-PI controller (*K*_*p*_ = 1200 and *K*_*i*_ = 60) while loaded with a mass of 8.04 g, showing **(A)** a controlled voltage input to the actuator (a combination of the proportional, integral and constant voltage terms of the controller) and **(B)** a height output with setpoint height, maximum height, rise time and settle time. Full zipping position occurs at zero height.

We used a set point height of 10 mm stroke away from the resting position to observe the performance of presented closed-loop control. Figure 6 shows closed-loop control of an electro-ribbon actuator under an external load of 8.04 g using the Boost-PI controller. Figure 6A shows the proportional and integral terms of the controller, the constant voltage *V*_*c*_ and the input voltage. Figure 6B shows the actuator response, setpoint, maximum height, rise time, and settle time.

The performance in controlling the actuator was explored by varying control gains of the Boost-PI controller. We varied *K*_*p*_ from 0 to 1,600 with increments of 200 and *K*_*i*_ from 0 to 60 with increments of 20 for each *K*_*p*_. *V*_*c*_ was set at 4,590 V (90% of *V*_*pull*−*in*_ for an actuator loaded with a mass of 8.04 g). Step-response control tasks (as shown in Figure 6) were performed for different control values; the experimental results can be concluded as follows:

- When *K*_*p*_ and *K*_*i*_ were both equal to zero (i.e., using only *V*_*c*_ without a PI controller), the actuator remained at the resting height without any zipping motion since the input voltage is equal to *V*_*c*_, which is less than *V*_*pull*−*in*_.
- When *K*_*i*_ = 0 (i.e., using the controller with only *K*_*p*_ and *V*_*c*_), when *K*_*p*_ was between 200 and 600, the actuator overshot the setpoint considerably, and zipped fully. With *K*_*p*_ ≥ 800, the proportional term was large enough to reduce applied voltage after overshoot quickly enough to prevent full zipping. With *K*_*p*_ ≥ 800, the actuator height approached the setpoint, although large steady-state error was present. Increasing *K*_*p*_ reduced the steady-state error but could negatively cause oscillation around the setpoint.
- When *K*_*p*_ = 0 (i.e., using the controller with only *K*_*i*_ and *V*_*c*_), the integral term slowly increased until the sum of the integral term and *V*_*c*_ exceeded *V*_*pull*−*in*_ to initialize actuation. At this point, full zipping occurred, because the integral term did not reduce applied voltage quickly enough to prevent full zipping.
- For any *K*_*i*_ (from 0 to 60), when *K*_*p*_ is 200, the actuator fully zipped (again, the proportional term was not large enough to reduce applied voltage after overshoot quickly enough to prevent full zipping). When *K*_*p*_ is 1400 and higher, unstable oscillations occurred.
- Using a controller with non-zero values of both *K*_*p*_, *K*_*i*_, and *V*_*c*_ allowed the actuator to converge to the setpoint at different velocities, when *K*_*p*_ was between 400 and 1,200 and *K*_*i*_ was between 20 and 60.

**Figure 7:**
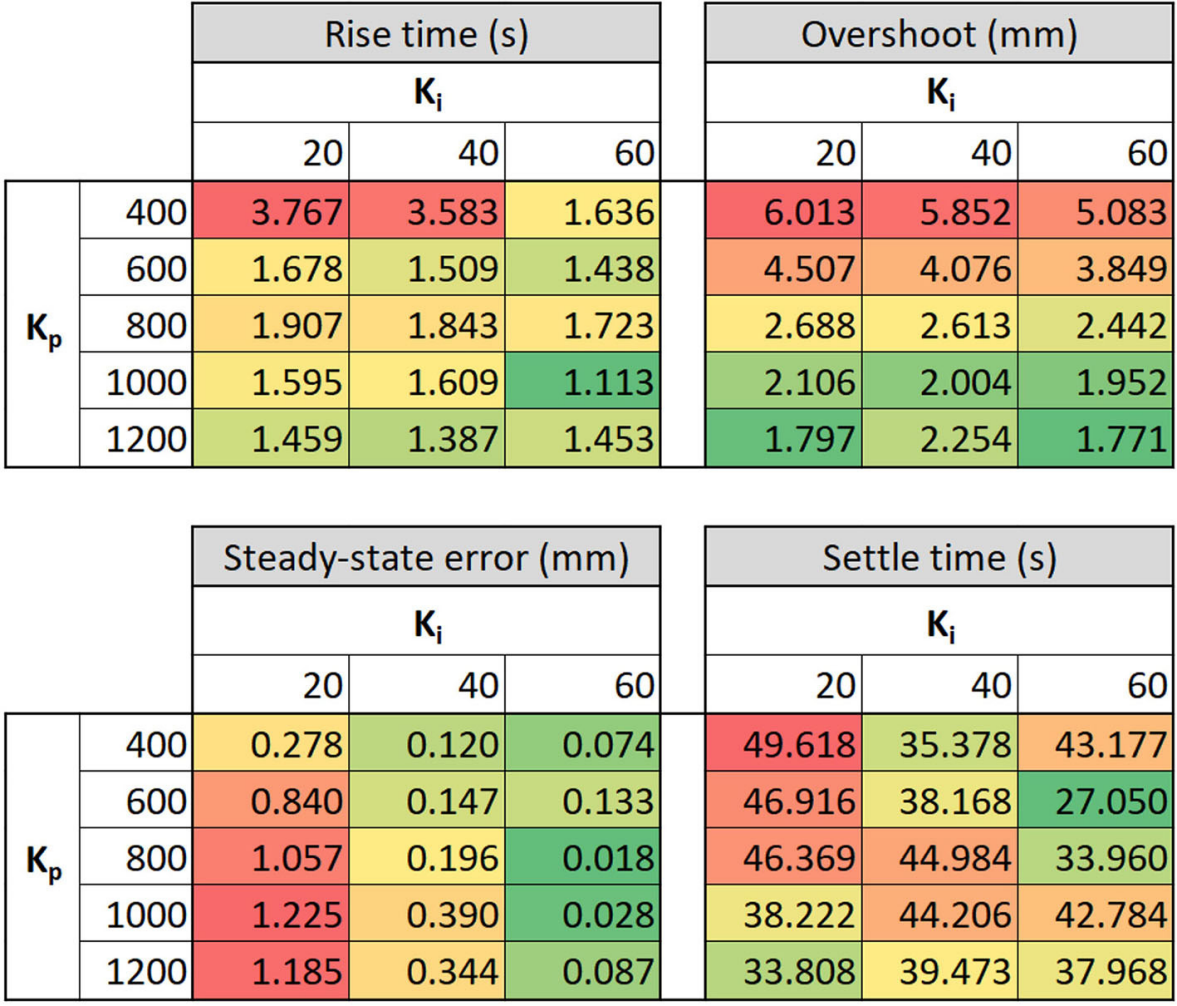
Key performance metrics of the electro-ribbon actuator when varying proportional gain *K*_*p*_ and integral gain *K*_*i*_: rise time, overshoot, steady-state error and settle time. Color scale is used to present the performance outcomes, where green and red colors indicate the best and the worst value, respectively.

The performance of the Boost-PI controller was evaluated by assessing performance metrics of steady state error, overshoot, rise time and settle time as benchmarks to select Boost-PI gains (Figure 7), considering only the case, where the actuator converges to set point position. Steady state error is defined as the difference between steady state height and setpoint height, while overshoot is the difference between steady state height and maximum height. Rise time is the time at which current height *h* reaches 90% of the setpoint height *h*_*s*_; settle time is the time at which *h* remains within 5% of the steady-state height.

According to Figure 7, rise time considerably decreased when *K*_*p*_≥ 600, down to between 1 and 2 s. Overshoot significantly decreased mainly by increasing *K*_*p*_ from 6.01 mm (*K*_*p*_= 400, *K*_*i*_= 20) to 1.77 mm (*K*_*p*_= 1,200, *K*_*i*_= 60), whereas steady-state error dramatically reduced with increasing *K*_*i*_ although it slightly increased with increasing *K*_*p*_. Settle time fluctuated across control gains, varying between 27 and 50 s with an average of 40 s. It is not possible to select gains which are ideal for all performance metrics, instead gains should be chosen that provide an appropriate compromise. In practice, the relative importance of each metric is problem-dependent and thus the Boost-PI gains should be selected according to the task at hand. This could be approached, for example, with a weighted-sum method using our performance metrics. Lower overshoot and faster rise time were prioritized for closed-loop control since lower overshoot enables higher controllable range close to full zipping position, and faster rise time increases the range of applications for this actuator. Hence, *K*_*p*_ = 1,200 and *K*_*i*_ = 60 were selected as task-appropriate general-use gains for this controller.

**Figure 8:**
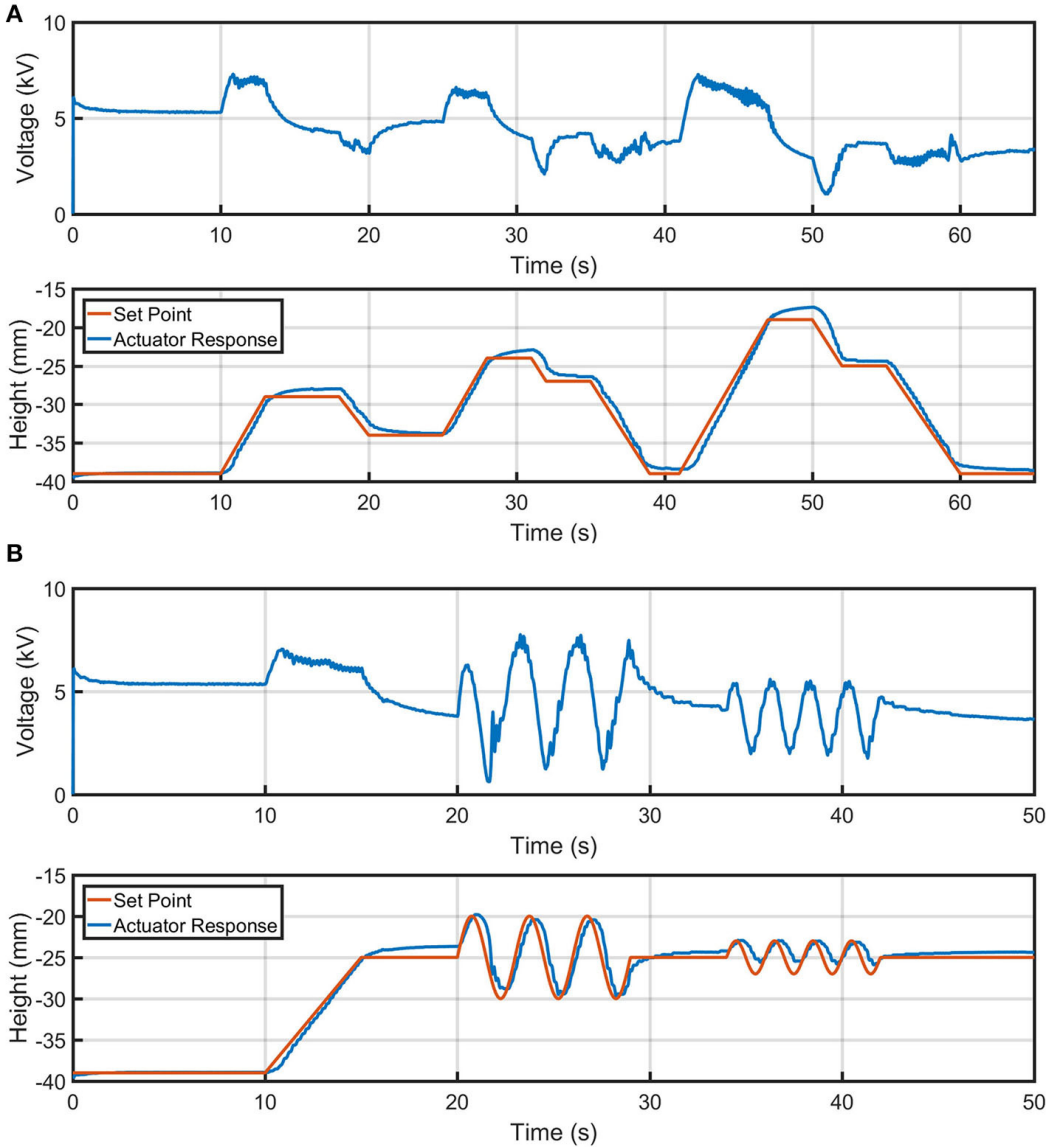
Controlled height output of an electro-ribbon actuator actuated using a closed-loop Boost-PI controller (*K*_*p*_ = 1,200 and *K*_*i*_ = 60) while loaded with a mass of 18.04 g, for different setpoint tracking tasks: **(A)** mountain and **(B)** sinewave. Full zipping position occurs at zero height.

We tested the versatility of the Boost-PI controller with two setpoint tracking tasks: a staircase task and an oscillatory task (Figure 8). *K*_*p*_ = 1,200 and *K*_*i*_ = 60 were used to control the actuator for these two tasks, which were set to perform within a 20 mm stroke range from a resting position to maintain high actuation performance (±10 mm stroke from where these control gains were analyzed). Comparing between the multiple cycles of sinusoidal set point control at 0.5 Hz as shown in Figure 8B, the maximum standard deviation for recorded actuator height between four repeated cycles was 0.5 mm. Although complex non-linearities clearly exist for the electro-ribbon actuator, it can be effectively controlled using the presented Boost-PI controller.

## Discussion

In this article, we explored an approach to control the contraction of an electro-ribbon actuator, which exhibits complex non-linear behavior. Initially, the actuator was tested by applying increasing static voltages to determine the *V*_*pull*−*in*_, where the actuator experienced pull-in instability and performed full zipping. *V*_*pull*−*in*_ depends on the external load; higher loads induce larger extensions and require higher *V*_*pull*−*in*_. If voltages below *V*_*pull*−*in*_ are applied, there exists a very narrow open-loop-controllable range of contractions. Application of a time-varying voltage approach, that is initially above *V*_*pull*−*in*_ but subsequently steps down to a lower voltage, enables a much wider range of accessible steady-state contractions. However, this approach is challenging because the steady-state contraction reached depends on not only on the applied voltage profile but also the previous steady-state contraction.

We modified a closed-loop PI controller—the Boost-PI controller—with an additional constant voltage term (*V*_*c*_) to control the actuator. *V*_*c*_ reduces the time taken for the integral term to ramp-up to the voltage required to initialize zipping, resulting in lower rise time. It ensured the controlled voltage was close to *V*_*pull*−*in*_, which is dependent to a suspended load (Figure 3B). The Boost-PI controller was studied by varying proportional and integral terms while setting *V*_*c*_ to 90% of *V*_*pull*−*in*_.

To select appropriate Boost-PI gains, we implemented a multi-objective parameter-space approach, analyzing rise time, overshoot, steady-state error and settle time of the actuator response as benchmarks. As a result, *K*_*p*_ = 1,200 and *K*_*i*_ = 600 were selected since the resulting actuator was capable of fast rise time (1.45 s or 5.89 mm/s), low overshoot (1.77 mm or 18.6%) and acceptable settle time (38.0 s) for a 9.49 mm setpoint distance step-response task. The Boost-PI controller was used to control an electro-ribbon actuator to perform different control tasks: a staircase and oscillatory task, showing versatility and controllability of electro-ribbon actuators.

The contraction rate of the electro-ribbon actuator increases with increasing supplied voltage (Taghavi et al., 2018). In contrast, its extension rate increases with decreasing supplied voltage and is lower than contraction due to stiction of dielectric liquid and surface tension between two electrodes. Applying high voltage results in high contraction speed but can result in full zipping. Using the presented PI controller enables the actuation speed up to 8.7 mm/s when using *K*_*p*_ = 1,000 and *K*_*i*_ = 60 and holds the actuator at stable height.

While our Boost-PI method demonstrates controllability of the electro-ribbon actuators, we note several limitations based on load, actuator length and the current height. At higher loads, increasing gravitational forces reduce the acceleration of the actuator when traveling upward (against gravity) and increase acceleration when traveling downward (with gravity) and would likely require retuning of controller gains. Furthermore, the sensitivity to variations in load increases when decreasing the bending stiffness of the actuator (a long beam is more sensitive due to the longer moment arm). An additional limitation of Boost-PI is set-point sensitivity. While we show good performance over a large range of travel for our actuator, electrostatic forces increasingly affect controller performance close to full zipping position.

To address these limitations, the gains could be configured to automatically respond to changes in load and height. While height dependent gains could be developed using feedback already present in the system, load dependency could be implemented with a load cell. This approach would allow electro-ribbon actuators to perform over a wider range of loads and set-points, decrease settle-time and mitigate the need to manually reconfigure gains. The electro-ribbon actuator was observed to access the minimum distance of 5 mm away from full zipping position (Figure 5). Automatically tuning control gains based on the current actuator shape could enable the actuator to access a smaller distance closer to the full zipping position.

Alternatively, to improve the actuator to counteract pull-in instability, the controllable range could be increased by implementing a direct charge control strategy that actively controls the level of electrostatic charge rather than voltage, as has been done for other MEMS devices (Bochobza-Degani et al., 2003; Zhang et al., 2014). Due to the novelty of electro-ribbon actuators, analytical models are not yet available, therefore we investigated the performance of our controller using a multi-objective parameter-space approach. We plan to use a model-based approach to control electro-ribbon actuators in future work.

Traditional closed-loop control using self-sensing has been demonstrated in electro-ribbon actuators over a small displacement range (Bluett et al., 2020). Using the capacitance of the actuator measured by a self-sensing unit, which increases with zipping, as a feedback variable for the proposed Boost-PI controller could result in a controlled electro-ribbon actuator without additional sensors, as required for many soft robotics applications.

The demonstrated Boost-PI controller enables closed-loop, high-accuracy, high-working-range displacement control of electro-ribbon actuators. This addresses one limitation of the electro-ribbon actuators and considerably extends the range of applications for this type of DLZ actuator, allowing it to be included in a wide range of soft robotic systems including wearables assist devices, autonomous rescue robots and soft robots for space exploration.

## Data Availability Statement

The datasets presented in this study can be found in online repositories. The names of the repository/repositories and accession number(s) can be found at: University of Bristol Research Data Repository (https://data.bris.ac.uk/data) at https://doi.org/10.5523/bris.2sv1xtrsmhoro2qu3io2u244ig.

## Author Contributions

RD and AF contributed equally throughout developing the presented controller, designing and performing experiments, and collecting and analyzing the data. TH, MT, and JR provided advices along the research project. All authors wrote the manuscript, read, and approved the submitted version.

## Conflict of Interest

The authors declare that the research was conducted in the absence of any commercial or financial relationships that could be construed as a potential conflict of interest.
